# Using the Internet for Health-Related Activities: Findings From a National Probability Sample

**DOI:** 10.2196/jmir.1035

**Published:** 2009-02-20

**Authors:** Nancy L Atkinson, Sandra L Saperstein, John Pleis

**Affiliations:** ^1^Department of Public and Community HealthPublic Health Informatics Research LaboratoryUniversity of MarylandCollege ParkMDUSA

**Keywords:** Internet, Web, health behavior, consumer

## Abstract

**Background:**

eHealth tools on the Internet have the potential to help people manage their health and health care. However, little is known about the distribution and use of different kinds of eHealth tools across the population or within population subgroups.

**Objective:**

The purpose of this study was to examine the prevalence and predictors of participation in specific online health-related activities.

**Methods:**

A secondary data analysis of the National Cancer Institute’s Health Information National Trends Survey (HINTS) 2005 was conducted to study three online behaviors among Internet users (n = 3244): searching for health information for oneself, participating in a support group for those with similar health or medical conditions, and purchasing medicine or vitamins.

**Results:**

A total of 58% of Internet users reported searching for health information for themselves, 3.8% used online support groups, and 12.8% bought medicine or vitamins online in the past year. Multivariate analysis found that those seeking health information were more likely to be women (OR = 2.23, 95% CI = 1.60, 3.09), have cable or satellite Internet connections (OR = 1.73, 95% CI = 1.22, 2.45) or DSL connections (OR = 1.94, 95% CI = 1.36, 2.76), have Internet access from work (OR = 2.43, 95% CI = 1.27, 4.67) or from home and work (OR = 1.73, 95% CI = 1.31, 2.30), and report more hours of weekday Internet use (OR = 4.12, 95% CI = 2.41, 7.07). Those with a high school education or less (OR = 0.44, 95% CI = 0.31, 0.63) and those with some college (OR = 0.66, 95% CI = 0.49, 0.89) were less likely to search for health information. Online support groups were more likely to be used by those with “fair” health (OR = 3.28, 95% CI = 1.21, 8.92) and “poor” health (OR = 5.98, 95% CI = 1.49, 24.07) and those with lower incomes (OR = 2.64, 95% CI = 1.09, 6.41) and less likely to be used by those with Internet access both at home and work (OR = 0.56, 95% CI = 0.35, 0.90). Those who were age 35-49 (OR = 2.16, 95% CI = 1.43, 3.26), age 50-64 (OR = 2.44, 95% CI = 1.53, 3.89), and age 65-74 (OR = 2.18, 95% CI = 1.30, 3.67) and those who were married (OR = 1.93, 95% CI = 1.13, 3.30) were more likely to purchase medicine or vitamins online.

**Conclusions:**

The Internet was most widely used as a health information resource, with less participation in the purchase of medicine and vitamins and in online support groups. Results suggest that modifying survey questions to better capture forms of online support and medications purchased could provide greater understanding of the nature of participation in these activities.

## Introduction

The purpose of this study was to examine the prevalence and predictors of participation in online health-related behaviors. Millions of people are now using the Internet on a regular basis, and much of this activity has been focused on health. Online eHealth tools have the potential to help people manage their health and health care, but little is known about the use of different kinds of eHealth tools across the population [1,2]. In 2003, an estimated 12.5 million online health-related searches were conducted globally each day [3]. However, aggregate figures like this do not tell us about what people are actually doing when they interact with online health-related content and tools. This study examined participation in three different online activities—health or medical information-seeking for oneself, use of online support groups for people with similar health and medical issues, and online purchase of medicine and vitamins—in order to better understand the potential of different health-related activities to influence people’s lives.

Understanding how people use the Internet for health-related activities can be critical to the design of relevant sites in order to attract and retain users [4]. One of the first steps in developing both traditional and computer-based health communication is an analysis of the target audience [5,6] to identify the audience’s preferences and needs and influence product acceptance and utilization [6]. Further, analysis of differential use of Internet applications by subpopulations can serve to identify potentially underserved groups and allow for the development of strategies to meet their needs [2].

Eysenbach [3] identified four broad applications of the Internet: (1) content (eg, information seeking), (2) community (eg, bulletin boards, chat rooms), (3) communication (eg, email), and (4) e-commerce (eg, buying or selling products and services on the Internet). The most commonly reported function for health has been content, or searching for health and medical information on the Internet [7]. Fewer Internet users have reported engaging in the other three functions for health-related purposes [7]. A recent study examined one health-related communication application—emailing a health care provider—and found a slow, but significant, increase in the prevalence of this activity from 2003 to 2005 (7% to 10% of Internet-using adults emailing a health care provider in the past year) [8]. The present study focused on health-related activities related to the other application areas of content, community, and e-commerce.

### Content

In 2000, 47% of Internet users reported that they looked for health information for themselves during their last online search [7]. Certain groups are more likely to search for health information: women [9,10] and those with higher education [9,10], a chronic health condition [10], more years of Internet experience [9], and broadband access [9]. The effect of income and age on online health information seeking has been inconsistent across studies [2,7,9,10]. No studies examining general Internet use have demonstrated racial/ethnic differences among those who seek health information online, but racial/ethnic differences have been found in the preferences and usage of content and community functions within specific online intervention and support programs [11-14]. For example, research examining the impact of a computer-based support system, the Comprehensive Health Enhancement Support System (CHESS), found that minority women with breast cancer spent more time using informational and decision-support features and were less likely to use the discussion groups than white women [11-13].

### Community

Few Internet users have reported contacting an online support group for a medical condition or personal problem—only 28% in 2001 [15]. At that time, women between the ages of 35 and 44 were more likely to participate in Internet groups to help manage daily responsibilities, including medical conditions. In 2005, participation in online chat rooms in general dropped to as low as 17% [16], which was attributed to a drop-off in women’s participation because of an increased concern about “worrisome” behavior in chat rooms.

### Commerce

Purchasing medications and vitamins online can be convenient and cost-effective [17], but this activity has occurred with low frequency thus far. In 2001, only about 5% of respondents had ever purchased prescription drugs online [18,19]. Three years later, only 4% of American adults reported ever purchasing drugs online [19]. However, one fourth (26%) of American adults reported looking on the Internet for information about prescription drugs in the same time period. Those with higher incomes and more years of Internet experience were more likely to purchase drugs online [19]. Most were satisfied with their purchase and reported that they would purchase drugs online again in the future, suggesting that the prevalence of this activity may increase.

These studies indicate that the use of the Internet for health reasons varies across population subgroups by the types of tools and services used and the frequency with which they are used. Ongoing research is needed to increase understanding about people’s preferences when using the Internet as a whole and to see how their use of these features changes over time. Further research is also needed to monitor activities, such as online purchase of medications [19]. Although few people are currently reporting participation in these activities, they have the potential to impact how people manage their health. Continued research can provide additional evidence about the nature of these online behaviors and determine whether their use will diffuse to a more general Internet-using population.

The present study was a secondary data analysis that examined the prevalence of specific health-related online activities and whether sociodemographic, health status, and Internet use factors were related to participation in these activities. The health-related Internet activities used in this study corresponded to Eysenbach’s application areas of content, community, and commerce [3]. Specifically, the research questions were (1) What is the prevalence of health or medical information seeking for oneself, use of online support groups for people with similar health and medical issues, and online purchase of medicine and vitamins? and (2) Are sociodemographic, health status, and Internet use differences related to participation in each of the three activities?

## Methods

This study analyzed data from the National Cancer Institute’s Health Information National Trends Survey (HINTS) 2005 to answer the research questions. The HINTS survey collects nationally representative data about the US public’s cancer communication practices, information preferences, risk behaviors, attitudes, and cancer knowledge [20]. The HINTS survey has been administered twice, first in 2003 and then again in 2005.

Participants in the 2005 survey (n = 5586) were recruited via random digit dialing of all telephone exchanges throughout the United States and then randomly selected from among the adults in the household. Survey administration averaged 30 minutes per participant. Response rates were 34% at the household screening level and 61% at the sampled person interview level [21].

All analyses were conducted using STATA Version 9 [22] to properly calculate standard errors for the multistage sampling design. The present analyses were conducted on the subset of the sample who reported going online, based on their response to the item “Do you ever go online to access the Internet or World Wide Web, or to send and receive e-mail?” All results were weighted to be representative of the United States adult population. To correctly calculate standard errors for the subset of adult Internet users residing in the United States, the SUBPOP statement in STATA Version 9 was used.

Descriptive statistics were calculated for all variables. Logistic regression analyses were conducted to answer the research question of whether selected sociodemographic, health status, and Internet use variables predict the Internet behaviors of looking for health information, participating in an online support group, or purchasing medicines or vitamins online.

HINTS questionnaire items were used to measure the following demographic variables: age, gender, ethnicity and race, education, income, marital status, number of children under 18 years in the home, general health status, and cancer history. Data for each variable were grouped into categories consistent with other research using HINTS data [23-25]. Additional items were selected in order to measure factors related to Internet use: location of access, type of connection, and frequency of use. Outcome variables measured use of the Internet for personal health information, use of online communities, and the purchase of medications or vitamins.

The unknown rates, which included missing, don’t know, or refused responses, were less than 4% for all variables except income. For all variables except income, unknowns were removed from the denominators when calculating percents. In the regression analyses, all unknowns, except those for income, were excluded from all of the models run and the subsequent results that are presented. Because of the relatively large number of respondents with unknown income, “unknown” was included in the regression analysis as a separate category. The HINTS questionnaire can be viewed at the National Cancer Institute’s website [26].

## Results

### Characteristics of the Sample Population

Over half (58%) of the sample, or 3244 respondents, reported that they used the Internet. The findings indicated that respondents were largely under age 50, non-Hispanic white, married, with some college education and annual incomes over US$50,000. The sociodemographic, health, and Internet use characteristics are summarized in Table 1.

Almost 90% of these Internet users reported Internet access at home, with 41% using a dial-up connection and almost 50% reporting high-speed connections. Over 60% of Internet users reported that they typically use the Internet for an hour on weekdays. These results suggest that using the Internet has become a ubiquitous part of daily life for many Americans. See Table 1 for a summary of Internet use characteristics.

**Table 1:** Sociodemographic, health, and Internet use characteristics of Internet users (n = 3244)

Characteristic	No.	Weighted Percent^a,b^
**Age**		
18-34	785	37.7
35-49	1076	33.3
50-64	958	22.1
65-74	301	5.0
75 and older	119	2.0
**Gender**		
Male	1169	48.2
Female	2075	51.8
**Race/ethnicity**		
Non-Hispanic white	2592	76.6
Non-Hispanic black	209	8.9
Hispanic	176	7.6
Non-Hispanic other	122	4.6
Multiple races	54	2.4
**Education**		
Less than high school	113	5.3
High school graduate	594	23.6
Some college	1029	38.3
Bachelor’s degree or higher	1430	32.8
**Household income**		
< $25,000	364	11.8
$25,000-$50,000	665	18.3
> $50,000	1770	55.7
Unknown	445	14.2
**Marital status**		
Married or living with a partner	2055	66.0
Divorced, widowed, or separated	584	9.7
Never married	521	24.3
**Household members under 18 years**		
None	1997	55.0
1 child	503	18.7
2 children	501	17.4
3 or more children	243	8.9
**General health status**		
Excellent	460	13.4
Very good	1157	33.3
Good	1070	37.0
Fair	405	13.8
Poor	85	2.6
**Cancer history: self**		
Yes	429	9.8
No	2812	90.2
**Location of access**		
Home only	1743	51.3
Home and other	179	9.1
Work only	195	4.9
Work and other	30	1.2
Home and work	856	24.4
Home, work, and other	76	2.9
Other location, not including home or work	161	6.0
**Type of Internet connection**		
Dial-up	1369	41.0
Cable or satellite	791	25.7
DSL	707	22.8
Other	254	7.1
**Weekday hours online**		
None	117	3.7
1 hour	2069	62.4
2 hour	527	17.4
3 hours	174	6.7
4 or more hours	245	9.9
**Weekend hours online**		
None	436	12.9
1 hour	1204	37.2
2 hour	666	20.2
3 hours	253	8.7
4 or more hours	576	20.9

^a^Results are weighted to be representative of the adult population of Internet users residing in the United States.

^b^Except for household income, unknown responses (“refused, “don’t know”) were excluded from the calculation of the percents shown in this table. The unknown rates for all characteristics besides household income were less than 4%.

### Prevalence of Health-Related Internet Activities

The most prevalent of the three studied activities was looking for health information for oneself, with 58.5% of respondents reporting that they had done so in the past 12 months. The other online activities occurred with lower frequency: 3.8% reported using an online support group for people with a similar health or medical issue, and 12.8% reported buying medicine or vitamins online.

### Determinants of Health-Related Internet Activities

Generally, each activity was predicted by a unique set of variables. Table 2 shows the results of the multivariate logistic regression analyses for each of the three online health activities.

**Table 2:** Multivariate logistic regression of predictors of participation in online health-related activities in the past 12 months (n = 3244)^a^

	Search for Health Information		Use Support Group		Buy Medicines or Vitamins	
	OR (95% CI)	*P*^b^	OR (95% CI)	*P*^b^	OR (95% CI)	*P*^b^
**Age**		.73		.28		.001
18-34	1.00		1.00		1.00	
35-49	1.13 (0.83, 1.54)		1.84 (0.82, 4.13)		2.16 (1.43, 3.26)^e^	
50-64	1.01 (0.73, 1.40)		1.64 (0.76, 3.52)		2.44 (1.53, 3.89)^e^	
65-74	0.92 (0.55, 1.53)		0.70 (0.13, 3.71)		2.18 (1.30, 3.67)^e^	
75 and older	0.75 (0.41, 1.38)		0.81 (0.19, 3.45)		1.65 (0.72, 3.81)	
**Gender**		< .001		.07		.23
Male	1.00		1.00		1.00	
Female	2.23 (1.60, 3.09)^e^		1.67 (0.96, 2.92)		1.21 (0.89, 1.64)^e^	
**Race/ethnicity**		.33		.98		.72
Non-Hispanic white	1.00		1.00		1.00	
Non-Hispanic black	0.72 (0.50, 1.04)		1.29 (0.34, 4.90)		1.25 (0.71, 2.20)	
Hispanic	1.02 (0.65, 1.61)		0.84 (0.19, 3.65)		0.92 (0.44, 1.94)	
Non-Hispanic other	0.83 (0.40, 1.70)		0.99 (0.34, 2.85)		0.82 (0.36, 1.87)	
Multiracial	1.58 (0.54, 4.64)		0.63 (0.06, 6.92)		1.84 (0.67, 5.01)	
**Education**		< .001		.06		.91
Bachelor’s degree and higher	1.00		1.00		1.00	
Some college	0.66 (0.49, 0.89)^e^		0.71 (0.40, 1.25)		0.98 (0.69, 1.38)	
High school or below	0.44 (0.31, 0.63)^e^		0.42 (0.20, 0.88)		0.91 (0.61, 1.37)	
**Household income**^c^		.15		.04		.39
$50,000 and higher	1.00		1.00		1.00	
$25,000-49,999	0.90 (0.64, 1.27)		1.32 (0.68. 2.58)		1.16 (0.73, 1.84)	
Less than $25,000	1.42 (0.96, 2.09)		2.64 (1.09, 6.41)^d^		1.11 (0.66, 1.86)	
**Marital status**		.77		.52		.03
Never married	1.00		1.00		1.00	
Married/living with partner	1.16 (0.76, 1.77)		1.66 (0.68, 4.05)		1.93 (1.13, 3.30)^d^	
Divorced/widowed/separated	1.12 (0.68, 1.84)		1.81 (0.57, 5.69)		1.47 (0.80, 2.74)	
**Household members < 18 years**		.01		.35		.14
None	1.00		1.00		1.00	
1 child	0.94 (0.68, 1.28)		0.60 (0.30, 1.20)		0.90 (0.61, 1.33)	
2 children	0.82 (0.53, 1.28)		0.78 (0.40, 1.51)		0.63 (0.40, 0.98)	
3 or more children	0.54 (0.38, 0.77)^e^		1.31 (0.59, 2.92)		0.57 (0.28, 1.16)	
**General health status**		.06		.01		.19
Excellent	1.00		1.00		1.00	
Very good	1.07 (0.74, 1.55)		1.27 (0.56, 2.89)		1.28 (0.84, 1.95)	
Good	1.39 (0.99, 1.94)		1.18 (0.49, 2.88)		1.03 (0.64, 1.66)	
Fair	1.42 (0.86, 2.33)		3.28 (1.21, 8.92)^e^		0.68 (0.38, 1.20)	
Poor	2.21 (0.61, 8.04)		5.98 (1.49, 24.07)^e^		1.27 (0.52, 3.07)	
**Cancer history: self**		.09		.34		.47
No	1.00		1.00		1.00	
Yes	1.39 (0.95, 2.03)		0.75 (0.42, 1.36)		0.86 (0.57, 1.30)^d^	
**Location of access**		.002		.05		.36
Home^f^	1.00		1.00		1.00	
Work^g^	2.43 (1.27, 4.67)^e^		2.28 (0.56, 9.19)		0.60 (0.25, 1.47)	
Home and work^h^	1.73 (1.31, 2.30)^e^		0.56 (0.35, 0.90)^d^		0.97 (0.72, 1.30)	
Other^i^	1.24 (0.57, 2.71)		1.38 (0.18, 10.45)		0.71 (0.19, 2.65)	
**Internet connection**^c^		.003		.68		.36
Dial-up	1.00		1.00		1.00	
Cable or satellite	1.73 (1.22, 2.45)^e^		0.98 (0.45, 2.12)		1.48 (0.95, 2.30)	
DSL	1.94 (1.36, 2.76)^e^		1.41 (0.74, 2.67)		1.46 (0.98, 2.19)	
Other	1.17 (0.66, 2.07)		0.49 (0.12, 2.05)		1.54 (0.70, 3.39)	
**Weekday hours online**		< .001		.08		.42
None	1.00		1.00		1.00	
1 hour	4.12 (2.41, 7.07)^e^		2.85 (0.25, 32.91)		1.78 (0.75, 4.23)	
2 hours	6.05 (3.18, 11.49)^e^		7.33 (0.56, 96.35)		2.15 (0.84, 5.52)	
3 hours	6.42 (2.53, 16.32)^e^		3.51 (0.24, 50.73)		2.75 (0.84, 8.98)	
4 or more hours	4.46 (1.56, 12.71)^e^		3.46 (0.22, 54.67)		1.79 (0.65, 4.96)	
**Weekend hours online**		.39		.42		.63
None	1.00		1.00		1.00	
1 hour	1.08 (0.69, 1.71)		2.03 (0.49, 8.44)		0.91 (0.56, 1.48)	
2 hours	1.25 (0.74, 2.10)		2.33 (0.48, 11.31)		0.93 (0.52, 1.66)	
3 hours	1.29 (0.64, 2.59)		1.05 (0.17, 6.44)		1.12 (0.58, 2.16)	
4 or more hours	1.79 (0.91, 3.55)		2.22 (0.35, 14.08)		1.28 (0.74, 2.21)	

^a^Results are weighted to be representative of the adult population of Internet users residing in the United States.

^b^ *P* values associated with Wald statistic.

^c^Analyses included unknown (“refused,” “don’t know”) responses that are not shown in the table.

^d^ *P* value is significant to the .05 level (*P* < .05).

^e^ *P* value is significant to the .01 level (*P* < .01).

^f^Includes “home only” and “home and other” responses.

^g^Includes “work only” and “work and other” responses.

^h^Includes “home and work only” and “home, work, and other” responses.

^i^Includes locations other than home or work.

#### Searching for Health Information for Oneself

Logistic regression analysis showed that gender, education, having children under age 18, location of Internet access, type of Internet connection, and hours of weekday Internet use predicted who was likely to search for health information. Women were more likely than men (OR [odds ratio] = 2.23, 95% CI [confidence interval] =1.60, 3.09) to search for health information. Those with a high school education or less (OR = 0.44, 95% CI = 0.31, 0.63) and those with some college education (OR = 0.66, 95% CI = 0.49, 0.89) were less likely to search for health information than those with a bachelor’s degree or higher. Those with three or more children under the age of 18 were less likely to look for health information for themselves than those with no children in the home (OR = 0.54, 95% CI = 0.38, 0.77).

Respondents with access from work (OR = 2.43, 95% CI = 1.27, 4.67) and access from home and work (OR = 1.73, 95% CI = 1.31, 2.30) were more likely to search for personal health information than those with primarily home access. Those with cable or satellite Internet connections (OR = 1.73, 95% CI = 1.22, 2.45) or DSL connections (OR = 1.94, 95% CI = 1.36, 2.76) were also more likely to search for personal health information than those with dial-up connections. Those who used the Internet for 1 hour or more on weekdays were more than four times as likely to search for health information as those who did not report daily Internet use.

#### Use of an Online Support Group

Health status, location of access, and income significantly predicted the use of online support groups for people with similar health or medical issues. Those who reported their health to be “fair” were 3.28 times as likely (95% CI = 1.21, 8.92) to use online support groups than those reporting their health to be “excellent.” Similarly, those with “poor” health were 5.98 times as likely (95% CI = 1.49, 24.07) to use online support than those in “excellent” health. Respondents with access from both home and work were about half as likely to use online support groups as those with access primarily from home (OR = 0.56, CI = 0.35, 0.90). Those with incomes lower than US$25,000 were more than twice as likely as those with incomes greater than US$50,000 to use online support groups (OR = 2.64, 95% CI = 1.09, 6.41).

#### Buying Medicines or Vitamins Online

Only two variables predicted the online purchase of medicines or vitamins: age and marital status. Adult Internet users ages 35-49, 50-64, and 65-74 were about twice as likely as users ages 18-34 to make these online purchases. Respondents who were married or living with a partner were almost twice as likely (OR = 1.93, 95% CI = 1.13, 3.30) as those who were never married to purchase medicines or vitamins online.

## Discussion

This study examined the prevalence of participation in online health activities and whether participation in these activities is predicted by sociodemographic, health status, and Internet use factors. We found that participation in these activities varied and that different sets of variables predicted who was likely to engage in these different activities.

Almost 60% of the Internet users surveyed reported searching for health information for themselves in the previous year. Fox and Rainie [7] identified the search for health information as the most common online health-related activity, and the present study found it to be the most prevalent of the three studied behaviors. This finding has several critical implications for both information providers and seekers. First, health agencies can use findings such as these to justify making health information available online because many people are using this channel, often before talking to their clinicians. Although people may prefer to go to their clinicians first, they have reported that they actually go online first for health information [27].

However, just making information available is not sufficient. Health agencies need to take responsibility for understanding how best to meet the needs of online information seekers. The rate of Internet use and broadband adoption has continued to increase among those with less than a high school education [28], suggesting that the number of users with lower literacy levels will grow [29]. Although this study found that respondents with higher education were more likely to search for health information, people with low literacy have identified health information as one of the primary types of information for which they would search [30]. Given that much of the health information on the Internet has been written at too high a level for many population segments [1], the development of materials that can be used and understood by audiences with lower literacy levels will be critical.

Another implication of online health information seeking relates to whether consumers can assess the quality of online health information. Despite the existence of reputable health portals, most users begin their search from a search engine [7] and rarely go beyond the first page of returned results [31]. Consumers must possess the skills to sort through and critically evaluate online information if the Internet is to realize its full potential in helping people meet their information needs.

Health status has been associated with health-related Internet use in previous studies [3,10], but these studies have reported conflicting evidence on whether having a chronic condition predicted health information seeking on the Internet. This study found that neither health status nor having been diagnosed with cancer were associated with greater Internet use for health information seeking. However, on the HINTS survey, respondents who indicated that they had been diagnosed with cancer could currently be in remission and not actively coping with a current cancer diagnosis. Thus, the use of this variable as a proxy measure of health status may not truly distinguish healthier versus less healthy individuals.

Health information seeking was also predicted by Internet use variables. Although almost all (90%) users had Internet access from home, this study found that those with access primarily from work or from both home and work were more likely to search for health information for themselves. People were more likely to use the Internet to search for health information if they used the Internet for an hour or more on typical weekdays and, as in previous research [9], had faster connections.

Having Internet access in the home has been considered an important indicator of equitable access among population groups [32], but the current results suggest that work access may be a critical factor in using the Internet for health information seeking. Frequency of use on weekdays and not weekends was related to searching for health information, again suggesting use on typical workdays. Accessing the Internet from work may offer some users faster connections. Given that most contact with medical professionals occurs during traditional work hours, receiving a phone call at work with a new diagnosis or lab results may provide a cue to action for an Internet search for those with the means to immediately begin searching. Those with access from both home and work may simply have more opportunities to search for health information.

The use of online support groups for people with a similar health or medical condition occurred with the lowest prevalence, with only 3.8% of participants reporting participation in such a group. Online support group use appears to be an infrequent activity, and the low rate of participation in health-related support groups may coincide with the general decline in online chat room participation reported by Fallows [16]. However, this finding may also result from the survey methodology. The HINTS survey question asked only about participation in online support groups; however, people may use Internet forums, bulletin boards, online communities, mailing lists, chat rooms, wikis, and blogs as means of providing or receiving online support or to share information. Users may not identify experiences with these different varieties of online communication as “participation in an online support group,” so this study may be underestimating the prevalence of Internet use for social support. Future surveys should first ask generally about use of the Internet for health-related social support and follow up with questions asking about specific mechanisms (eg, message boards, blogs) for getting support.

The present study found that those reporting “fair” or “poor” health were more likely to use online support groups than those with “excellent” health. This suggests that those with greater health needs appear to be taking greater advantage of the Internet to help them cope with their conditions, even though online support groups usage was limited overall.

Those with lower incomes were also more likely to use online support groups, although previous studies found that those with higher incomes were more likely to search for health information [10] or purchase medications online [19]. Possibly, those with higher incomes have other means of support, while those with lower incomes are turning to the Internet for assistance. An implication of this finding may be that people with limited incomes are replacing medical care with online support groups, which may put them at risk if they do not get needed assessments and treatment. However, this finding could also be the result of statistical error based on the low rate of participation in this activity overall as well as the high number who did not report their income.

Respondents with Internet access only from their home were more likely to use online support groups than those with access at both home and work, suggesting that those who were “less connected” may have greater need for online support. Continued research on the impact of location of Internet access and use of online social support tools is needed to understand and validate this relationship.

Almost 13% of respondents reported purchasing medications or vitamins online, which is much higher than the 4% and 5% reported in previous studies [18,19] and the 9% in the 2003 HINTS survey [33]. Given the potential for negative outcomes if poor oversight and regulation of this activity occur, research on the impact of the online purchase of medications and vitamins could be expanded so that we better understand the nature of this behavior. In contrast to Fox’s [19] finding that income and years of Internet experience predicted online medication purchase, this study found only age and marital status to be significant predictors. This activity may have increased recently because of national discussions about prescription drugs and increased interest in complementary and alternative medicine. It may also reflect the increase in use of the Internet for e-commerce in general [34].

Across online health-related activities, this study examined the relative importance of specific sociodemographic variables. One of the variables most connected with these activities was gender. The current research found that women were more likely than men to engage in a search for health information for themselves. They were equally as likely as men to participate in online support groups and to purchase medications and vitamins. However, married participants were more likely to purchase medications and vitamins online, indicating that women may have additional influence on this behavior.

Traditionally, men have been less likely than women to engage in health information seeking and preventive health behaviors. Since men do use the Internet for so many other activities, perhaps the Internet provides an opportunity to provide non-traditional health education opportunities directed toward men. A comparative content analysis of existing websites for men’s and women’s health is needed to determine gaps in such resources. Continued formative research could also help determine the types of health-related sites or online formats that would better appeal to male audiences.

Despite implications from other Internet intervention studies [11-14], racial and ethnic minorities appeared to engage in health-related activities at rates similar as white users. These results may suggest that use of the World Wide Web is different than use of Internet-based systems on which these studies have been conducted. Even though African American and Hispanic populations have long shown lower rates of Internet adoption as compared to white users [32,35,36], the current research suggests that once online, they are as likely as white users to engage in health-related activities. Ongoing research is needed to understand the patterns of use by different subpopulations.

### Limitations

This study was limited by its examination of only three select online activities. People may be engaging in many other online activities, such as searching for health information for others, using online behavior change or disease management programs, locating a health care provider, or researching health insurance plans. The Internet also offers several new ways to interact with other people via social media not captured by the current HINTS survey. Further examination of these additional activities would provide a larger window into the use of the Internet for health purposes. A second limitation of this study was that it only included data from those who were online. Comparing those who are online to those who are offline could further identify subgroup differences and differential information needs.

This study was also limited by nature of the HINTS data. The response rate, which has declined from the 2003 HINTS survey, may mean that systematic differences exist between those who responded and those who did not. In addition, the data are based on self-report, which can be biased by social desirability.

### Conclusion

In conclusion, this study found that, in the context of health, the Internet is still most widely used as an information resource, with much less use for the purchase of medications and vitamins and participation in online support groups. As the types of tools and activities available to Internet users expand, the tools to monitor their use must also expand. Modifying and adding survey items would enable better measurement of Internet participation, especially in online support and social media mechanisms that could not be determined in the current research. Added items would also reveal the types of medications and vitamins people obtained online and show whether additional consumer safety and patient education information needs exist.

This study found that having access to the Internet from both home and work increased the likelihood of searching for personal health information and decreased the likelihood of participating in online support groups, indicating the need to understand more about the impact of work access. Future studies using HINTS data could determine the importance of other predictive variables. For example, these data could be used to examine whether people who use online support groups are less likely to be employed or if people who are employed are less likely to use support groups overall. This kind of research has the potential to distinguish the role of employment status from having access to the Internet at work.

Women continue to be the most likely audience for health-related online activities, while racial and ethnic minorities had rates higher than expected. Further research should be conducted into strategies to reach other audiences or to determine which channels may better serve those who do not use the Internet for health. Now that HINTS has been administered in 2003 and 2005 and another administration is being completed, future research will be possible to identify trends in participation in these and new online health behaviors.

## Abbreviations

HINTS: Health Information National Trends Survey

## References

[1] US Department of Health and Human Services (2006). Expanding the reach and impact of consumer e-health tools.

[2] Lorence Daniel P, Park Heeyoung, Fox Susannah (2006). Assessing health consumerism on the Web: a demographic profile of information-seeking behaviors. J Med Syst.

[3] Eysenbach Gunther (2003). The impact of the Internet on cancer outcomes. CA Cancer J Clin.

[4] (2004). Drug company’s consumer health portal encourages return visits. Internet Healthc Strateg.

[5] US Department of Health and Human Services (2002). Making Health Communication Programs Work.

[6] Kreps Gary L (2002). Evaluating new health information technologies: expanding the frontiers of health care delivery and health promotion. Stud Health Technol Inform.

[7] Fox S, Rainie L (2000). The online health care revolution: how the Web helps Americans take better care of themselves.

[8] Beckjord Ellen Burke, Finney Rutten Lila J, Squiers Linda, Arora Neeraj K, Volckmann Lindsey, Moser Richard P, Hesse Bradford W (2007). Use of the internet to communicate with health care providers in the United States: estimates from the 2003 and 2005 Health Information National Trends Surveys (HINTS). J Med Internet Res.

[9] Fox S (2005). Health information online.

[10] Bansil Pooja, Keenan Nora L, Zlot Amy I, Gilliland Jeanne C (2006). Health-related information on the Web: results from the HealthStyles Survey, 2002-2003. Prev Chronic Dis.

[11] Gustafson DH, Hawkins R, Pingree S (2001). Effect of Computer Support on Younger Women with Breast Cancer. J Gen Int Med.

[12] Gustafson David H, Hawkins Robert P, Boberg Eric W, McTavish Fiona, Owens Betta, Wise Meg, Berhe Haile, Pingree Suzanne (2002). CHESS: 10 years of research and development in consumer health informatics for broad populations, including the underserved. Int J Med Inform.

[13] McTavish FM, Pingree S, Hawkins R, Gustafson D (2003). Cultural differences in use of an electronic discussion group. J Health Psych.

[14] Rimer Barbara K, Lyons Elizabeth J, Ribisl Kurt M, Bowling J Michael, Golin Carol E, Forlenza Michael J, Meier Andrea (2005). How new subscribers use cancer-related online mailing lists. J Med Internet Res.

[15] Horrigan JB, Rainie L (2001). Online communities: networks that nurture long-distance relationships and local ties.

[16] Fallows D (2005). How women and men use the Internet.

[17] Wen H Joseph, Tan Joseph (2005). Mapping e-health strategies: thinking outside the traditional healthcare box. Int J Electron Healthc.

[18] Baker Laurence, Wagner Todd H, Singer Sara, Bundorf M Kate (2003). Use of the Internet and e-mail for health care information: results from a national survey. JAMA.

[19] Fox S (2004). Prescription drugs online.

[20] Nelson David E, Kreps Gary L, Hesse Bradford W, Croyle Robert T, Willis Gordon, Arora Neeraj K, Rimer Barbara K, Viswanath K V, Weinstein Neil, Alden Sara (2004). The Health Information National Trends Survey (HINTS): development, design, and dissemination. J Health Commun.

[21] Cantor D, Covell J, Davis T, Park I, Rizzo L Health Information National Trends Survey 2005 Final Report.

[22] (2005). Stata Statistical Software: Release 9.

[23] Han Paul K J, Moser Richard P, Klein William M P (2006). Perceived ambiguity about cancer prevention recommendations: relationship to perceptions of cancer preventability, risk, and worry. J Health Commun.

[24] Rutten LJ, Squiers L, Hesse B (2006). Cancer-related information seeking: Hints from the 2003 Health Information National Trends Survey (HINTS). J Health Comm.

[25] Squiers L, Bright MA, Rutten LJ (2006). Awareness of the National Cancer Institute’s Cancer Information Service: Results from the Health Information National Trends Survey (HINTS). J Health Comm.

[26] Health Information National Trends Survey: Search HINTS Questions.

[27] Hesse Bradford W, Nelson David E, Kreps Gary L, Croyle Robert T, Arora Neeraj K, Rimer Barbara K, Viswanath Kasisomayajula (2005). Trust and sources of health information: the impact of the Internet and its implications for health care providers: findings from the first Health Information National Trends Survey. Arch Intern Med.

[28] Horrigan J (2006). Home Broadband Adoption.

[29] Nielsen J Lower-literacy users.

[30] Zarcadoolas C, Blanco M, Boyer JF, Pleasant A Unweaving the web: an exploratory study of low-literate adults’ navigation skills on the world wide web. J Health Comm Jul-Sep;7(4):309-24..

[31] Morahan-Martin Janet M (2004). How internet users find, evaluate, and use online health information: a cross-cultural review. Cyberpsychol Behav.

[32] US Department of Health and Human Services (2000). Healthy People 2010. 2nd edition. With Understanding and Improving Health and Objectives for Improving Health. 2 vols.

[33] National Cancer Institute.

[34] US Department of Commerce (2004). A Nation Online: Entering the Broadband Age.

[35] US Department of Commerce (1999). Falling Through the Net: Defining the Digital Divide.

[36] Fox S (2005). Digital Divisions.

